# Alginate Hydrogels with *Aloe vera*: The Effects of Reaction Temperature on Morphology and Thermal Properties

**DOI:** 10.3390/ma15030748

**Published:** 2022-01-19

**Authors:** Katarzyna Bialik-Wąs, Konstantinos N. Raftopoulos, Krzysztof Pielichowski

**Affiliations:** 1 Department of Organic Chemistry and Technology, Faculty of Chemical Engineering and Technology, Cracow University of Technology, 24 Warszawska Str., 31155 Cracow, Poland; 2 Department of Chemistry and Technology of Polymers, Faculty of Chemical Engineering and Technology, Cracow University of Technology, 24 Warszawska Str., 31155 Cracow, Poland; konstantinos.raftopoulos@pk.edu.pl (K.N.R.); kpielich@pk.edu.pl (K.P.)

**Keywords:** hydrogels, sodium alginate/poly (vinyl alcohol) matrix, *Aloe vera*, DSC analysis

## Abstract

In this study, we investigated the impact of reaction temperature on the physicochemical, structural, morphological, and thermal properties of sodium alginate/poly (vinyl alcohol)-based hydrogels, both in the pure form and with the addition of 20% (*v*/*v*) *Aloe vera* solution. The materials were prepared by chemical crosslinking at temperatures in the range of 65–75 °C. Poly (ethylene glycol) diacrylate was used as a crosslinking agent. The extent to which the crosslinking reaction proceeded was studied as a function of the reaction temperature, along with the thermal properties and morphology of the final materials. A measurement of gel fraction, in agreement with differential scanning calorimetry and Fourier transform infrared spectroscopy, showed that a higher temperature of reaction promoted the crosslinking reaction. On the basis of the aforementioned techniques, as well as by energy dispersive X-ray analysis under an electron microscope, it was also shown that the bioadditive *Aloe vera* promoted the crosslinking reaction.

## 1. Introduction

Hydrogels based on natural polymers have attracted a great deal of attention from the fundamental research community, in a bidirectional relationship with their increasing use in medical, cosmetic, and pharmaceutical applications [1,2,3]. Gelatin, starch, chitosan, collagen, hyaluronic acid, dextran, glucan, and alginate are the most commonly utilized polymers. We may distinguish different classes of hydrogels with respect to their polarity, e.g., nonionic, anionic, cationic, amphoteric, and zwitterionic [4,5,6,7,8,9]. With respect to their physical structure, they can be amorphous, semicrystalline, hydrogen-bonded, supramolecular, or hydrocolloidal, depending also on their crystallinity or lack thereof [10,11]. Moreover, hydrogels may be characterized by their composition, for instance homopolymeric, copolymeric, IPNs (interpenetrating polymer networks), and semi-IPNs [12,13].

The preparation of hydrogels can be achieved via physical or chemical crosslinking [14,15,16]. However, the specific chemical or physical approach should be adapted to the base polymers and the potential applications of the synthesized hydrogels. In the case of physical crosslinking, the following methods have been used: freeze-thawing (poly (vinyl alcohol) (PVA), PVA/chitosan); stereocomplex formation (poly(l-lactide)-poly(d-lactide)); ionic interaction (chitosan, alginates); H-bonding (hyaluronic acid); and maturation (heat-induced aggregation, hyaluronic acid) [16,17,18,19,20,21,22,23,24,25,26,27]. Meanwhile, chemically crosslinked hydrogels can be obtained using chemical or radiation grafting (poly (ε-caprolactone), poly (ethylene glycol), *N*-vinyl caprolactam, chitosan); radical polymerization (poly (ethylene glycol) methyl ether methacrylate); condensation or enzymatic reaction (Β-cyclodextrin, chitosan); UV, microwave, gamma, or electron beam irradiation (poly (vinyl methyl ether), alginate/PVA, chitosan, polyethylene oxide (PEO), and poly (acrylic acid) (PAAc), polyvinylpyrrolidone (PVP)/carboxymethyl cellulose (CMC)) [16,28,29,30,31,32,33,34,35,36,37]. The choice of the synthetic route for hydrogel preparation is significant because the type of crosslinking method used may affect the final properties [20]. By understanding the processes and choosing the appropriate method, it is possible to prepare modern and smart polymeric materials with targeted action and precisely defined properties. In order to obtain hydrogels with high mechanical strength and a high degree of crosslinking, chemical crosslinking methods are preferred. Moreover, these matrices, in the context of drug delivery systems and other medical applications, allow the release of active substances in a sustained and controlled manner. In contrast, in the case of ionic crosslinking, the release of the drug is faster [20], which is desirable in certain applications, such as in the cosmetic industry, where it may be a useful property from the point of view of the therapeutic activities. Depending on the crosslinking method, a varied morphology of hydrogels can be obtained [38,39]. Their structure is characterized by high porosity, especially in the case of physical crosslinking, such as freeze-thawing or ionic interaction.

The final properties of hydrogel products are primarily dependent on the monomers, polymers, and crosslinking agents used. Consequently, natural or/and synthetic polymers that are highly biocompatible, biodegradable, and non-toxic as well as safe for humans and animals, such as chitosan, gelatin, alginates, collagen, poly (vinyl alcohol), poly (ethylene oxide) (PEG), and hyaluronic acid, are commonly applied in the preparation of hydrogels [39,40,41,42,43].

In the course of our work on polymers as drug delivery vehicles, we synthesized a hydrogel system with potential applications where biocompatibility and biodegradability as well as good drug delivery properties are desired [20,34,35]. It is based on the naturally derived-isolated from marine brown algae, sodium alginate (SA), crosslinked with poly (ethylene glycol) diacrylate (PEGDA). Sodium alginate was chosen due to its wide availability, biocompatibility, biodegradability, and non-toxicity. Poly (vinyl alcohol) (PVA) was added to the system in order to provide better mechanical properties, such as strength, elastic modulus, strain, toughness, and flexibility, in a dry state, while at the same time being suitable for biomedical applications because of its biocompatibility, nontoxicity, and non-carcinogenicity. *Aloe vera* extract, a mixture of several active substances, was also introduced as a natural healing agent. Finally, the introduction of glycerin into the system was expected to improve the transdermal permeability of active substances.

The proposed SA/PVA-based multifunctional hydrogels were studied in our previous articles [20,34,35] using a higher reaction temperature, such as 80 °C. However, in the context of the further modification of the basic matrix, we took into account that some active substances, i.e., enzymes, antibiotics, and proteins, are thermolabile [44,45,46]. In order to decrease the possibility of the loss of their biological activity in this study, we investigated materials prepared at lower temperatures. More specifically, we studied the impact of reaction temperature on the degree of crosslinking and the swelling ratio of the resulting hydrogels. Based on this investigation, we showed that *Aloe vera* seems to influence the progress of the reaction. This study was complemented by an investigation of the thermal transitions of the substrates and the final hydrogels. This provided information on both the progress of the reaction in an indirect manner and the characterization of the final properties as a function of the reaction temperature.

## 2. Materials and Methods

### 2.1. Materials

Sodium alginate and poly (ethylene glycol) diacrylate (PEGDA) Mn = 700 g/mol (used as a crosslinking agent) were purchased from Sigma-Aldrich (Darmstadt, Germany). Poly (vinyl alcohol) (M_n_ = 72,000 g/mol), ammonium persulphate (employed as an initiator), and glycerin were acquired from Avantor Performance Materials Poland S.A. (Gliwice, Poland). *Aloe vera* lyophilisate was purchased from a local shop with cosmetics and herbal raw materials (Zrób Sobie Krem, Kraków, Poland).

### 2.2. Methods

#### 2.2.1. Preparation of Hydrogel Materials

**Scheme 1:**
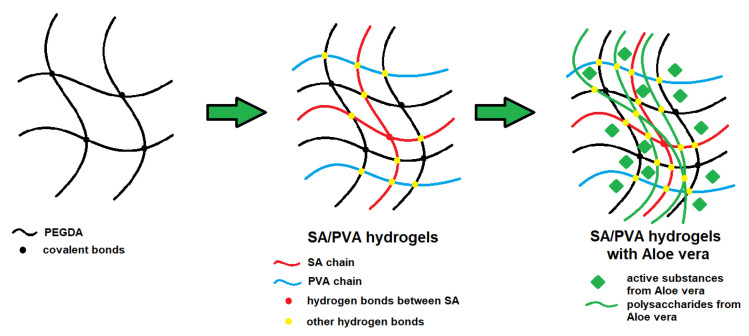
The potential interactions between SA, PVA, *Aloe vera*, and PEGDA.

To obtain the hydrogel materials, stock aqueous solutions were prepared, namely a 5% solution of poly (vinyl alcohol) (PVA), 2% solution of sodium alginate (SA), 2% solution of *Aloe vera* lyophilisate, and 1% solution of ammonium persulfate. Next, the stock solutions of PVA and SA were mixed in a 1:1 *v*/*v* ratio along with constant amounts of poly (ethylene glycol) diacrylate (7.5%, *v*/*v*) and glycerin (1.7%, *v*/*v*). In the case of *Aloe vera* modified materials, 20% *v*/*v* of the *Aloe vera* stock solution was also added. Subsequently, the mixtures were heated to 70 °C and then 4.4% (*v*/*v*) of ammonium persulfate was added. After that, all reaction mixtures were poured into Petri dishes and placed on a heating plate at three different temperatures, 65, 70, and 75 °C, for 1.5 h. Finally, the materials were placed in ambient conditions for 24 h [47]. The potential interactions between alginates, PVA chains, *Aloe vera*, and PEGDA are presented in Scheme 1.

#### 2.2.2. Determination of Gel Fraction

Three pieces of approximately 10 × 10 mm were cut from each hydrogel, conditioned at a temperature of 40 °C for 24 h, and weighed (w_0). Then, they were immersed in distilled water at room temperature for 48 h up to an equilibrium swelling weight. After that, the gel materials were conditioned again at 40 °C for 24 h and weighed (w_e). The gel fraction (%GF) was calculated as:

%GF = w_e/w_0 × 100%(1)

The reported results are the averages of the three specimens cut out from each hydrogel.

#### 2.2.3. Determination of Swelling Behavior

The swelling ratio was determined by immersion in PBS (phosphate buffer) solution (Chempur, Piekary Śląskie, Poland) (pH = 7.4) and distilled water at ambient temperature. First, samples were conditioned at 40 °C for 24 h, and their mass (w_d) was recorded. The samples were then immersed in fluids. The swollen samples were removed and weighed (w_s) at time intervals 5, 15, 30, 60, and 1440 min. Each hydrogel was tested in triplicate. The swelling ratio of all the tested hydrogel samples was determined as (2):

%SR = (w_s-w_d)/w_d × 100%(2)

The reported results are the averages of the three specimens cut out from each hydrogel.

#### 2.2.4. Fourier Transform Infrared Spectroscopy (FTIR)

The chemical structure of the hydrogels was studied with a Thermo Scientific Nicolet iS5 FT-IR spectrometer equipped with an iD7 ATR accessory (Waltham, MA, USA) in the range of 4000−400 cm^−1^. All spectra (32 scans at 4.0 cm^−1^ resolution) were recorded at 25 °C.

#### 2.2.5. Scanning Electron Microscopy (SEM)

Scanning electron microscopy was conducted with a JSM-6010LA microscope equipped with an energy-dispersive X-ray spectroscopy (EDS) detector (Tokyo, Japan). The surface of the as-received hydrogel films was coated with gold with nominal thickness of 4 nm and imaged in a vacuum. No special drying procedure was undertaken, beyond the aforementioned equilibration at ambient conditions.

#### 2.2.6. Differential Scanning Calorimetry (DSC)

The thermal transitions of the hydrogels were studied using differential scanning calorimetry (DSC). The pure components were also studied for comparison. The experiments were performed using a Mettler Toledo 822 calorimeter (Warsaw, Poland), calibrated with indium, cooled with liquid nitrogen, and purged with argon. Solid or liquid samples of 7–10 mg were enclosed in sealed aluminum pans (Mettler-Toledo, Warsaw, Poland) and subjected to the following thermal protocols: for hydrogels/PEGDA/glycerin, cooling to −100 °C, heating to 135 °C (first heating), cooling to −100 °C, heating to 180 °C (second heating); for PVA, two heating–cooling cycles from room temperature to 25 °C; for sodium alginate, two heating–cooling cycles from −120 °C to 135 °C; and for *Aloe vera*, one heating from −120 °C to 180 °C. All heating/cooling steps were performed at rate 10 K/min.

The glass transition temperatures were calculated at midpoint.

## 3. Results

### 3.1. Gel Fraction

**Figure 1:**
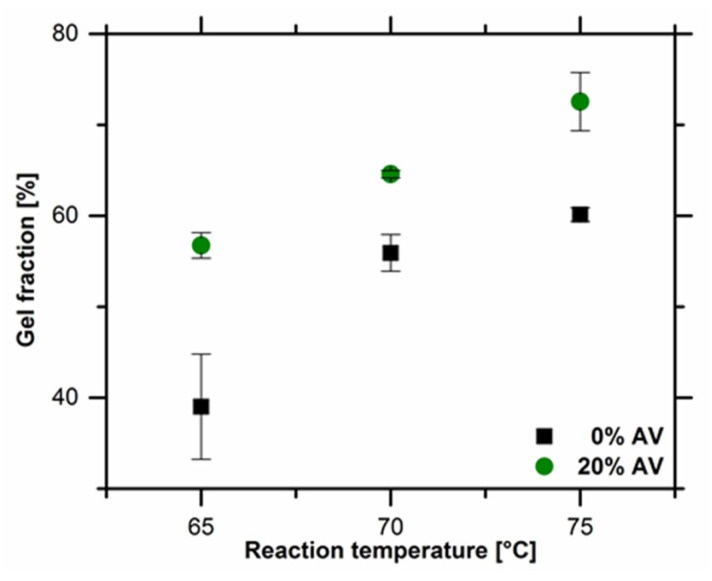
The gel fraction of obtained hydrogel materials.

The gel contents in SA/PVA hydrogels with and without *Aloe vera* are presented in Figure 1 as a function of reaction temperature.

Lower values of gel fraction were obtained for samples without *Aloe vera* solution, which resulted from the viscosity of the reaction mixture. A measurement with a rotary viscosimeter (Brookfield, Toronto, ON, Canada) showed that the reaction mixture without *Aloe vera* had a viscosity of 0.026 ± 0.001 Pa·s at 25 °C while the mixture with *Aloe vera* had a viscosity of 0.533 ± 0.011 Pa·s. Most likely, this dependence is associated with the presence of additional polysaccharides and mucopolysaccharides from *Aloe vera*. A similar dependence was observed in previous studies [35] when samples contained different amounts of *Aloe vera*, and the differences in the gel fractions ranged from 60 to 70%. Moreover, an increasing trend of the gel fractions with increasing crosslinking temperature from 65 to 75 °C was observed. This result clearly suggests that the reaction temperature has a key role in the extent of the chemical crosslinking, with higher temperatures resulting in more crosslinked materials. For this reason, the selection of the proper reaction temperature depends on the type of polymeric mixture used.

### 3.2. Swelling Ability

**Figure 2:**
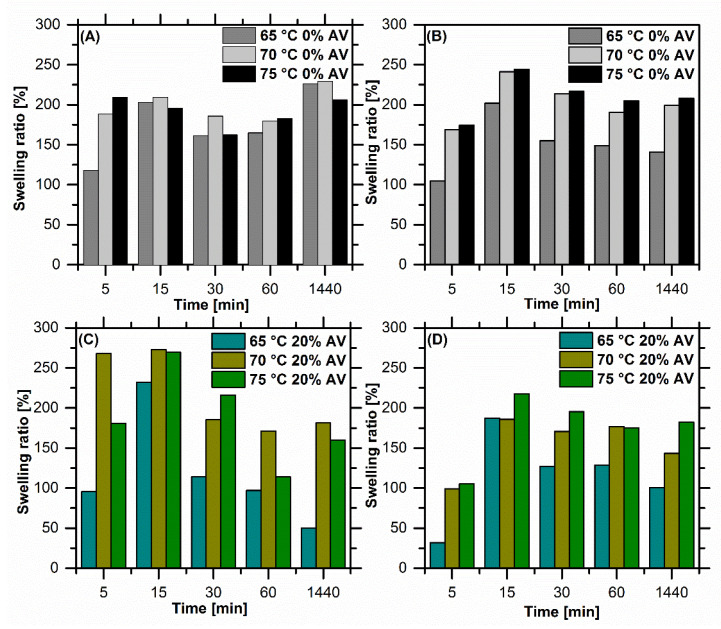
Swelling ratio (%) in (**A**,**C**) distilled water and (**B**,**D**) PBS for SA/PVA hydrogels: (**A**,**B**) without and (**C**,**D**) with *Aloe vera*. Note that the *x*-axis is not linear.

The swelling abilities of SA/PVA hydrogels in distilled water and PBS before and after modification with *Aloe vera* obtained using different reaction temperatures are presented in Figure 2.

Hydrogels can absorb large amounts of water and other fluids in a reversible manner. However, the extent of this property depends on the composition as well as the crosslinking method. The swelling degrees in Figure 2 showed that the tested samples behaved similarly in both distilled water and PBS solution. The highest swelling values were in the range of approximately 200–275%, which is consistent with the literature [48]. Nevertheless, in the case of the samples containing *Aloe vera*, the swelling ratio in water was slightly higher than in PBS. This likely originates from the release of active substances from the pH-sensitive matrix, which allows this process to take place in the slightly alkaline (pH = 7.4) environment of PBS [49].

It is noteworthy that for all analyzed hydrogel samples, it was observed that the swelling ratio did not increase monotonically with time; rather, it reached a maximum at the early stages, mainly around the 15 min point. This effect has been observed in the literature [50]. This phenomenon is especially visible for samples containing *Aloe vera*. The swelling ratio was then almost 275%. Upon this maximum, the swelling ratio decreased slowly, an effect that was especially noticeable in PBS. This may be attributed to the expansion of the SA chains, which leads to the breakdown of the crosslinking bonds by the presence of the PO_4_^3−^ anion in the medium [51]. As a consequence, part of the polymer may dissolve in the aqueous medium, effectively leading to a decrease in the mass of the polymer.

The lowest swelling abilities were observed for the samples that were obtained at temperature of 65 °C. This likely resulted from a weakly crosslinked matrix, because the gel fraction was only 39.00 ± 5.79%. Moreover, the porosity of hydrogels directly influences their swelling capacity. The literature [52] clearly indicates that large hydrogel pores enable better contact with water molecules, which leads to a higher swelling ratio. However, in the case of smaller pores, the structure of hydrogels is more dense and compact. Hence, their water uptake is higher.

### 3.3. Fourier Transform Infrared Spectroscopy (FTIR)

**Figure 3:**
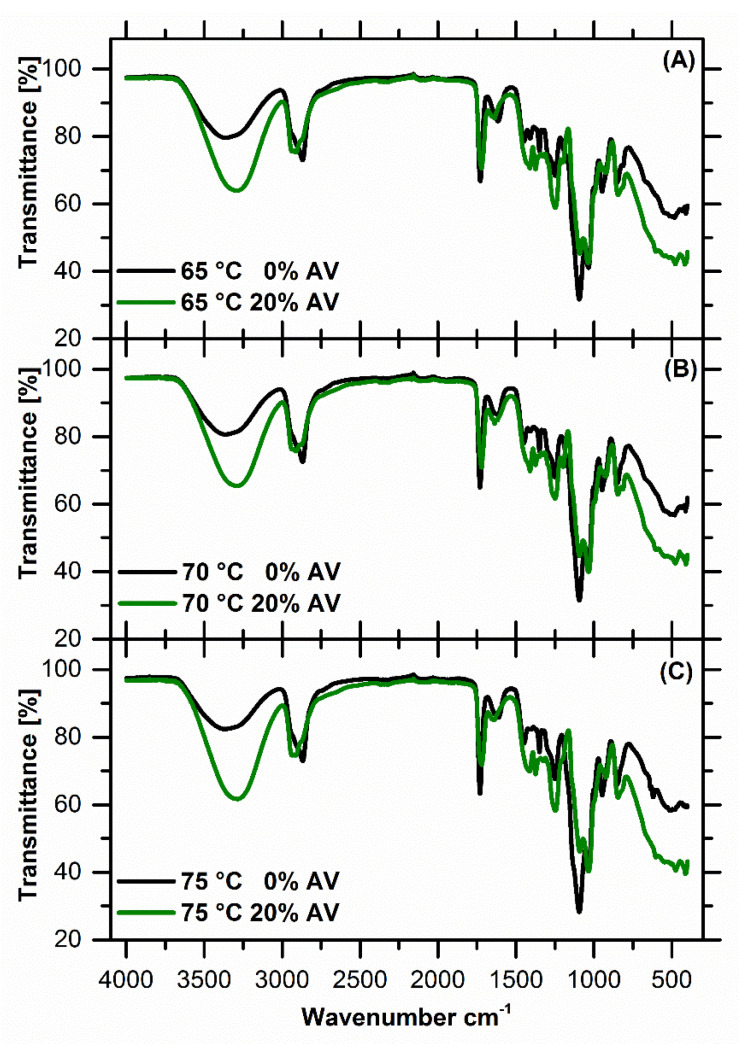
FTIR spectra of hydrogels before and after modification with *Aloe vera*, obtained at temperatures of (**A**) 65 °C, (**B**) 70 °C, and (**C**) 75 °C.

FTIR spectra of SA/PVA hydrogels in pure form and modified with *Aloe vera*, which were obtained using a different reaction temperature, i.e., 65, 70, or 75 °C, are presented in Figure 3.

In the range of 3500–3000 cm^−1^ a broad band was observed, which is characteristic of stretching vibrations of the O-H groups coming from several components of the polymeric composition, i.e., sodium alginate (SA), poly (vinyl alcohol) (PVA), and additionally from *Aloe vera*. This band was more intensive in all samples containing 20% AV because of the presence of polysaccharides and stronger hydrogen bonds [35]. The stretching vibrations of C-H groups occur at a wavenumber of 2940 cm^−1^. Additionally, at 1035 cm^−1^ we observed vibrations from the C–O and C–OH bonds in glucan units present in the *Aloe vera* polysaccharide as well as in the alginate molecules. Referring to the chemical structure of alginates, the peaks at 1607 and 1450 cm^−1^ correspond to the asymmetric and symmetric stretching vibrations of carboxylate anion (COO−), respectively. The bands appearing at 1250 cm^−1^ and 1030 cm^−1^ were assigned to C-O-C in glycosidic bonds. Moreover, we observed bands located at 985 and 810 cm^−1^ that were attributed to COH out-of-plane bending and -CH_2_ twisting. In all spectra, vibrations appeared at 1730 cm^−1^, indicative of ester groups from the PEGDA. Moreover, the stretching vibrations of C-O-C group in PEGDA occur at 1164, 1190, and 1035 cm^−1^ [35,53,54,55].

Interestingly, when the crosslinking reaction was conducted for the mixtures containing *Aloe vera*, especially at 75 °C, the vibrations coming from -C=C bonds were less visible and they were shifted. This is in par with the observation that the gel fraction was high for this material, and the assumption that the crosslinking reaction was more complete.

### 3.4. Differential Scanning Calorimetry (DSC)

**Figure 4:**
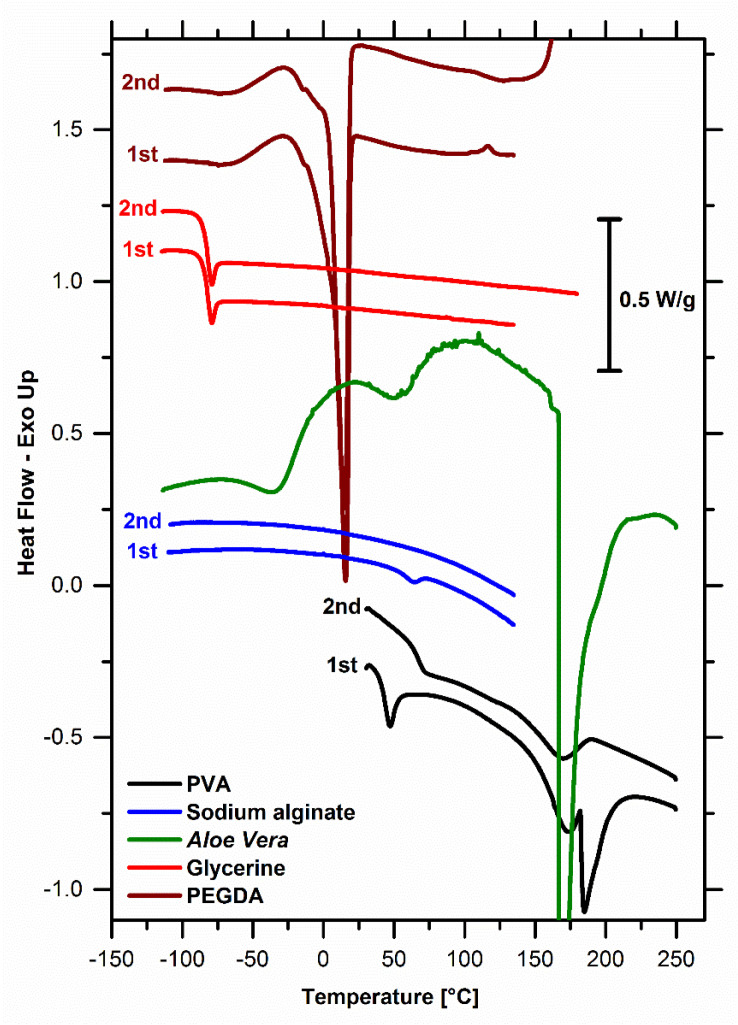
Heating DSC curves of the substrates. The curves have been translated for clarity.

The DSC curves of the substrates are shown in Figure 4. Starting with PEGDA, the main feature was a strong melting endotherm around 18 °C, in agreement with the literature [56]. This melting endotherm was preceded by what appeared to be a cold crystallization exotherm. No clear glass transition step was observed, possibly because it was masked by the nearby cold crystallization. A kink around −15 °C has been interpreted by Ghadimi et al. [56] as a glass transition step; however, we consider this as part of the melting process. It should be mentioned that the detection of the glass transition of PEG is not an easy task due to its strong crystallinity, especially in its dry form, and values in the range of −80 to −70 °C are reported in the literature [57,58] for slightly hydrated systems.

The other major component of the system, PVA, showed a clear glass transition step at lower temperatures accompanied during the first run by an enthalpy relaxation endothermic peak. The glass transition temperature increased considerably between the two runs, likely as a result of different degrees of crystallinity and/or evaporation of water. At temperatures above 130 °C, we observed peaks associated with the melting of the polymer. Interestingly, the as-received material exhibited two peaks, possibly due to the existence of two types of crystallites, while in the second run only one broad peak was observed.

Sodium alginate did not exhibit any considerable effects, except for a weak endotherm around 65 °C, presumably attributed to melting. *Aloe vera* exhibited two endothermic peaks around −35 and 50 °C, probably due to the melting of two of its components. At 160 °C, a very strong endotherm corresponds to its degradation.

**Figure 5:**
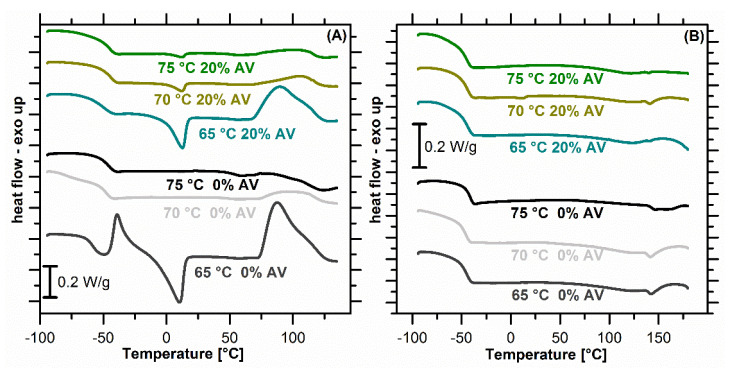
Heating thermograms of the hydrogels: (**A**) first heating; (**B**) second heating. Labels indicate the reaction temperature.

We now turn our attention to the hydrogels. During the first heating (Figure 5A) at low temperatures, a step corresponding to the glass transition was observed around −50 °C. We will discuss its dependence on composition and reaction temperature later in the text.

The materials reacted at 65 °C, both with and without *Aloe Vera*, exhibited an endothermic peak around 10 °C, which was attributed to the melting of unreacted PEGDA. Interestingly, for the material without *Aloe vera* prepared at 65 °C, melting was preceded by a cold crystallization endotherm, which indicated that crystallization in this system was not completed during cooling, either due to slow kinetics or to the significant supercooling needed for nucleation. The intensity of the melting peak is a measure of the extent of the crosslinking reaction, as reacted PEGDA chains are less likely to align and crystalize within the network. In that respect, the decrease in its intensity with reaction temperature is in agreement with the gel fraction and FTIR results. The fact that it occurred at all temperatures for the *Aloe vera*-containing networks may signify that this additive promoted nucleation of the basic polymer.

At higher temperatures, starting around 70 °C, a complex exothermic peak appeared, associated with the crosslinking reaction occurring in the system. This peak was very intense for the materials reacted at 65 °C, indicating that at this temperature the reaction proceeded very slowly and was not completed in the assigned time. This is further evidence that higher temperatures promoted the reaction. It is also worth mentioning that no endotherms that could be associated with water evaporation were visible in the DSC curves, except maybe for the unmodified material produced at 75 °C.

We now turn our attention to Figure 5B, and the second heating runs. Here, no exotherms associated with the crosslinking reaction appeared, indicating that the process was completed. Interestingly, the melting peaks vanished too, in agreement with the assumption that the extent of the reaction would be negatively correlated to the intensity of the melting peak. The latter option is corroborated by the trend observed in the first run, namely that water melting was suppressed with increased reaction temperature (and thus a higher degree of crosslinking).

At higher temperatures, in the region of 80–150 °C, there was a very broad endotherm that could be associated with an order–disorder transition of an origin that is not clear at this point. Superimposed on the broad endotherm, there was a narrow endotherm that was associated with the melting of PVA, which had migrated to lower temperatures. This suppression of melting temperature is associated with the development of smaller crystallites, as the semicrystalline polymer is restricted topologically in the complex network.

**Figure 6:**
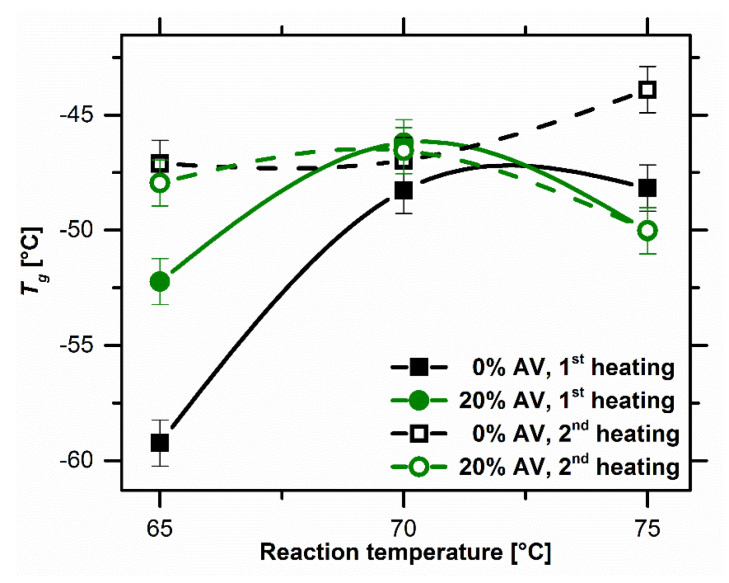
Glass transition temperature as a function of reaction temperature.

Glass transition temperature Tg, calculated at midpoint, as a function of reaction temperature is shown in Figure 6 for both heating runs. The values were somewhat elevated as compared to the values for PEG reported in the literature [57,58], which is a result of the reduction in constraints imposed by crosslinking as well as copolymerization with the much less mobile PVA. The suppression of crystallinity due to crosslinking allows the now amorphous segments to exhibit a well-defined glass transition step.

Indeed, the incompletely crosslinked systems synthesized at the low temperature of 65 °C had the lowest glass transition temperatures; however, due to the increased degree of crosslinking for materials prepared at 70 °C, and the corresponding restrictions of mobility the glass transition temperature increased. For the formulation lacking *Aloe vera*, the glass transition temperature did not increase further with a reaction temperature of 75 °C. Surprisingly, for the aloe-containing material, the glass transition temperature decreased by a few degrees. The reason behind this observation should be a mechanism that increases mobility when the reaction is performed at elevated temperatures. This mechanism is unclear at this point.

The glass transition temperature increased in the second run for the materials prepared at 65 °C, as a result of the completed crosslinking reaction. The effect was more pronounced in the unmodified hydrogel. As mentioned earlier, the *Aloe vera* formulation seemed to have a higher degree of crosslinking than in the initial materials. The materials prepared at 70 °C did not exhibit any considerable difference. However, the unmodified material prepared at 75 °C presented a noticeable increase, which could be associated with the endotherm of yet unclarified origin that was observed at 120 °C during the first heating run.

### 3.5. Scanning Electron Microscopy (SEM)

**Figure 7:**
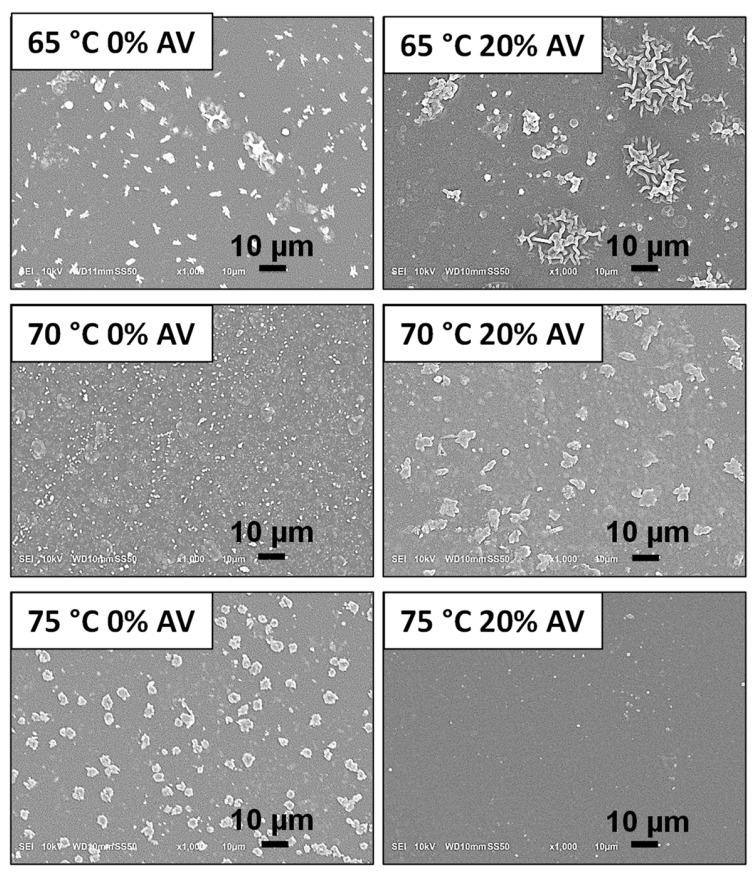
SEM micrographs presenting the morphology of obtained hydrogels before immersion in PBS solution.

Figure 7 shows SEM micrographs recorded of the surface of all the materials under investigation. The surface of the hydrogels was very smooth, uniform, without pores, and similar for all analyzed samples. These results are consistent with the data from the literature [59,60]. However, some agglomerates were present in all the materials, with the exception of the hydrogel containing *Aloe vera* that was obtained using the highest reaction temperature (75 °C).

**Figure 8:**
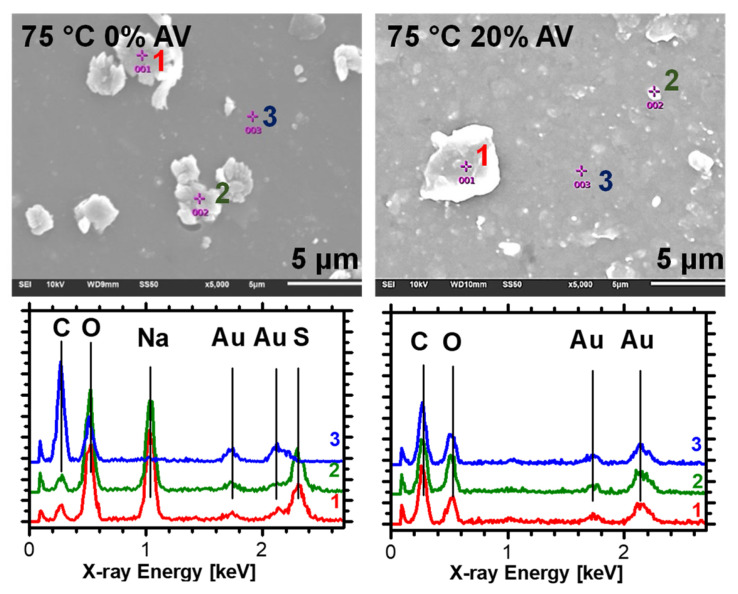
SEM-EDS analysis of hydrogels obtained using a temperature of 75 °C, before and after modification with *Aloe vera*.

In order to shed some light on the nature of those aggregates, EDS point measurements of hydrogels were conducted (Figure 8) on both hydrogels prepared at 75 °C. The measurements confirmed the presence of the following elements on the surface of the sample without *Aloe vera*: C, O, Na, Au, and S. For the hydrogel containing *Aloe vera*, only C, O, and Au appeared. It is apparent that the gold came from the sputtering of the samples before measurement. The presence of sulfur can be associated with the unreacted initiator—ammonium persulfate. It was also noted that sodium appeared, which was derived from the basic polymer—sodium alginate. Most likely, the polymer was not properly crosslinked; this is why the sodium alginate was found on the surface of the analyzed sample. In this situation, it is possible to refer to the gel fraction. When a lower reaction temperature (65 °C) was used, the gel fraction was quite weak at about 40 and 58% for the hydrogel without and with *Aloe vera*, respectively. Using a higher temperature (75 °C), a higher gel fraction was obtained, which is visible for the samples containing *Aloe vera*.

**Figure 9:**
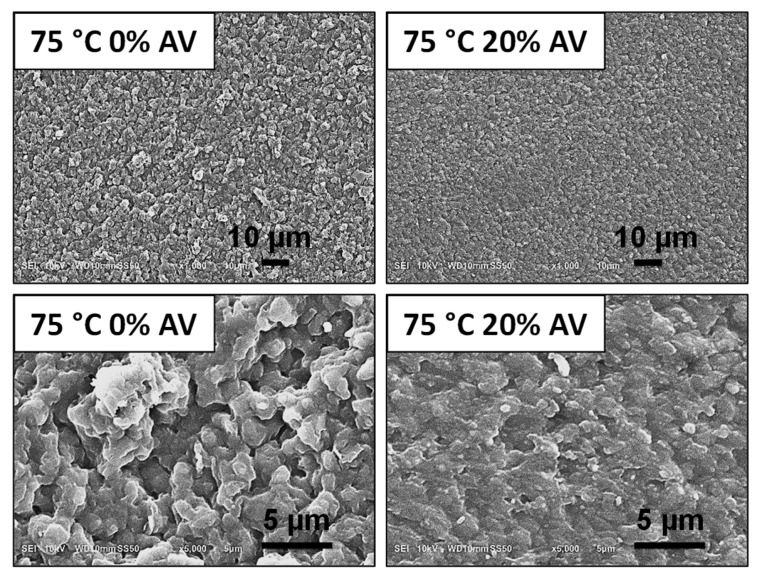
SEM micrographs presenting the morphology of hydrogels obtained using a temperature of 75 °C; samples were analyzed after immersion in PBS solution for 24 h.

**Figure 10:**
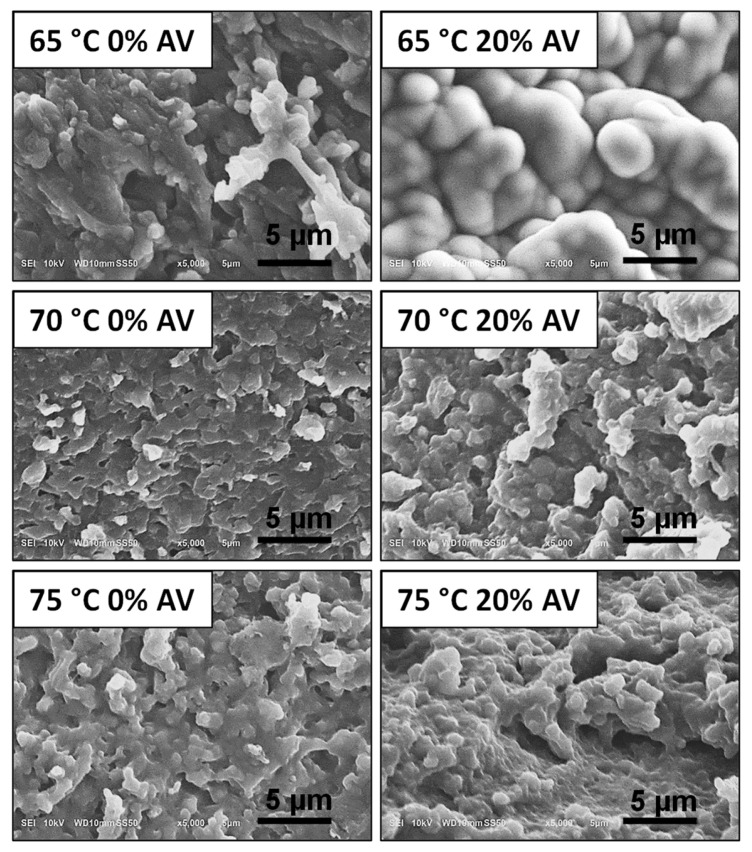
SEM micrographs show the cross-section of obtained hydrogels.

In the next step, the morphology and cross-sections of selected hydrogels were tested after immersion in PBS solution for 24 h (Figure 9 and Figure 10).

This analysis showed that the surface was more varied and irregular. Moreover, the morphology of the samples without *Aloe vera* (75_0AV) was characterized by slightly larger spaces between the polymeric chains than in the case of the modified hydrogels (75_20AV) [35]. The presence of *Aloe vera* in the system causes more efficient progress of the crosslinking reaction, which was also confirmed based on the gel fraction results [60]. Additionally, the surface of the samples with *Aloe vera* was more compact and less porous, which could result from many active substances, such as mucopolysaccharides and polysaccharides, which penetrate into the free spaces of the crosslinked polymer.

The cross-sections of the hydrogel samples proved the porous 3D structure and exhibited multilayered internal networks in the matrix. It was estimated that the average pore size was less than 5 µm. Moreover, when we looked at the cross-sections deeply, we could also see that, in the case of aloe-containing hydrogel samples, the polymer networks were arranged closer to each other. In addition, the morphology was more irregular and less porous, which resulted from the presence of many the active substances derived from *Aloe vera*. This is shown in Figure 9.

## 4. Conclusions

In summary, the obtained results proved that the reaction temperature is a crucial parameter during hydrogel preparation using the sol casting method. Additionally, it was shown that the presence of *Aloe vera* in the reaction mixture had a positive impact and significantly improved the gel fraction content up to 72.56 ± 3.21% by using a reaction temperature of 75 °C. This was also confirmed by the FT-IR spectra and DSC curves. The physicochemical analysis of swelling abilities indicated that the tested hydrogels behave similarly in both distilled water and PBS solution, and the highest swelling ratios were in the range of approximately 200–275%. However, the lowest swelling values were determined for the samples that were obtained using a temperature of 65 °C, which was caused by a very weakly crosslinked matrix. Moreover, the SEM images of the morphology showed that the surface of the obtained was very smooth, uniform, without pores, and similar for all analyzed hydrogels. However, the cross-section of these materials showed that they were characterized by a porous structure, which created multi-layered three-dimensional networks. In the case of the hydrogels modified with *Aloe vera*, the surface was more compact and less porous, which may have resulted from the additional content of mucopolysaccharides and polysaccharides that penetrate into the free spaces of the crosslinked polymer. SEM-EDS analysis indicated that the use of lower reaction temperatures was not sufficient, as evidenced by the observed presence of sulfur from the unreacted initiator, ammonium persulfate.

## Data Availability

The data that support the findings of this study are contained within the article.

## References

[1] Varghese S.A., Rangappa S.M., Siengchin S., Parameswaranpillai J. (2019). Natural polymers and the hydrogels prepared from them. Hydrogels Based on Natural Polymers.

[2] Olatunji O. (2016). Classification of Natural Polymers. Natural Polymers Industry Techniques and Applications.

[3] Rajeswari S., Prasanthi T., Sudha N., Swain R.P., Panda S., Goka V. (2017). Natural polymers: A recent review. World J. Pharm. Sci..

[4] Gyles D.A., Castro L.D., Carréra Silva J.O., Ribeiro-Costa R.M. (2017). A review of the designs and prominent biomedical ad-vances of natural and synthetic hydrogel formulations. Eur. Polym. J..

[5] Saini K. (2017). Preparation method, properties and crosslinking of hydrogel: A review. PharmaTutor.

[6] Kirchmajer D.M., Gorkin Iii R., Panhuis M.I.H. (2015). An overview of the suitability of hydrogel-forming polymers for extrusion-based 3D-printing. J. Mater. Chem. B.

[7] Yoshida R., Okano T., Ottenbrite R.M., Park K., Okano T. (2010). Stimuli-Responsive Hydrogels and Their Application to Functional Materials. Biomedical Applications of Hydrogels Handbook.

[8] Garg S., Garg A. (2016). Hydrogel: Classification, properties, preparation and technical features. Asian J. Biomater. Res..

[9] Mahinroosta M., Farsangi Z.J., Allahverdi A., Shakoori Z. (2018). Hydrogels as intelligent materials: A brief review of synthesis, properties and applications. Mater. Today Chem..

[10] Khansari M.M., Sorokina L.V., Mukherjee P., Mukhtar F., Shirdar M.R., Shahidi M., Shokuhfar T. (2017). Classification of Hydrogels Based on Their Source: A Review and Application in Stem Cell Regulation. JOM.

[11] Van Vlierberghe S., Dubruel P., Schacht E. (2011). Biopolymer-Based Hydrogels as Scaffolds for Tissue Engineering Applications: A Review. Biomacromolecules.

[12] Muhammad Z., Waqar S., Sadaf W., Rai M.S., Asif M., Junaid Q., Javed I., Fazal R.S., Muhammad S.R., Usman K. (2015). Hy-drogels, their applications and polymers used for hydrogels: A review. Int. J. Biol. Pharm. Allied Sci..

[13] Nilimanka D. (2013). Preparation methods and properties of hydrogel: A review. Int. J. Pharm. Pharm. Sci..

[14] Slaughter B.V., Khurshid S.S., Fisher O.Z., Khademhosseini A., Peppas N.A. (2009). Hydrogels in Regenerative Medicine. Adv. Mater..

[15] Chung H.J., Park T.G. (2009). Self-assembled and nanostructured hydrogels for drug delivery and tissue engineering. Nano Today.

[16] Varaprasad K., Raghavendra G.M., Jayaramudu T., Yallapu M.M., Sadiku R. (2017). A mini review on hydrogels classification and recent developments in miscellaneous applications. Mater. Sci. Eng. C.

[17] Figueroa-Pizano M., Vélaz I., Peñas F., Zavala-Rivera P., Rosas-Durazo A., Maldonado-Arce A., Martínez-Barbosa M. (2018). Effect of freeze-thawing conditions for preparation of chitosan-poly (vinyl alcohol) hydrogels and drug release studies. Carbohydr. Polym..

[18] Shi X., Wu J., Wang Z., Song F., Gao W., Liu S. (2020). Synthesis and properties of a temperature-sensitive hydrogel based on physical crosslinking via stereocomplexation of PLLA-PDLA. RSC Adv..

[19] Liu S., Oderinde O.K., Hussain I., Yao F., Fu G. (2018). Dual ionic cross-linked double network hydrogel with self-healing, conductive, and force sensitive properties. Polymer.

[20] Bialik-Wąs K., Królicka E., Malina D. (2021). Impact of the Type of crosslinking agents on the properties of modified sodium algi-nate/poly(vinyl alcohol) hydrogels. Molecules.

[21] Ye X., Li X., Shen Y., Chang G., Yang J., Gu Z. (2017). Self-healing pH-sensitive cytosine-and guanosine-modified hyaluronic acid hydrogels via hydrogen bonding. Polymer.

[22] Erickson I.E., Kestle S.R., Zellars K.H., Dodge G.R., Burdick J.A., Mauck R.L. (2012). Improved cartilage repair via in vitro pre-maturation of MSC-seeded hyaluronic acid hydrogels. Biomed. Mater..

[23] Harada A., Kobayashi R., Takashima Y., Hashidzume A., Yamaguchi H. (2010). Macroscopic self-assembly through molecular recognition. Nat. Chem..

[24] Sun T.L., Kurokawa T., Kuroda S., Ihsan A.B., Akasaki T., Sato K., Gong J.P. (2013). Physical hydrogels composed of polyam-pholytes demonstrate high toughness and viscoelasticity. Nat. Mater..

[25] Grindy S., Learsch R.W., Mozhdehi D., Cheng J., Barrett D.G., Guan Z., Messersmith P., Holten-Andersen N. (2015). Control of hierarchical polymer mechanics with bioinspired metal-coordination dynamics. Nat. Mater..

[26] Ke H., Yang L.-P., Xie M., Chen Z., Yao H., Jiang W. (2019). Shear-induced assembly of a transient yet highly stretchable hydrogel based on pseudopolyrotaxanes. Nat. Chem..

[27] Huang Z., Chen X., O’Neill S.J.K., Wu G., Whitaker D.J., Li J., McCune J.A., Scherman O.A. (2021). Highly compressible glass-like supramolecular polymer networks. Nat. Mater..

[28] An Y.M., Liu T., Tian R., Liu S.X., Han Y.N., Wang Q.Q., Sheng W.J. (2015). Synthesis of novel temperature responsive PEG-b-[PCL-gP (MEO2MA-co-OEGMA)]-b-PEG (tBG) triblock-graft copolymers and preparation of tBG/graphene oxide composite hydrogels via click chemistry. React. Funct. Polym..

[29] Cruz A., García-Uriostegui L., Ortega A., Isoshima T., Burillo G. (2017). Radiation grafting of N-vinylcaprolactam onto nano and macrogels of chitosan: Synthesis and characterization. Carbohydr. Polym..

[30] Wang J., Wei J. (2016). Hydrogel brushes grafted from stainless steel via surface-initiated atom transfer radical polymerization for marine antifouling. Appl. Surf. Sci..

[31] Zhao H., Gao J., Liu R., Zhao S. (2016). Stimulus-responsiveness and methyl violet release behaviors of poly(NIPAAm-co-AA) hydrogels chemically crosslinked with β-cyclodextrin polymer bearing methacrylates. Carbohydr. Res..

[32] Ebrahimi M.-M.S., Voss Y., Schönherr H. (2015). Rapid Detection of Escherichia coli via Enzymatically Triggered Reactions in Self-Reporting Chitosan Hydrogels. ACS Appl. Mater. Interfaces.

[33] Chaykar A.S., Goharpey F., Yeganeh J.K. (2016). Volume phase transition of electron beam cross-linked thermo-responsive PVME nanogels in the presence and absence of nanoparticles: With a view toward rheology and interactions. RSC Adv..

[34] Bialik-Wąs K., Pluta K., Malina D., Majka T. (2019). Alginate/PVA-based hydrogel matrices with *Echinacea purpurea* extract as a new approach to dermal wound healing. Int. J. Polym. Mater..

[35] Bialik-Wąs K., Pluta K., Malina D., Barczewski M., Malarz K., Mrozek-Wilczkiewicz A. (2020). Advanced SA/PVA-based hydrogel matrices with prolonged release of Aloe vera as promising wound dressings. Mater. Sci. Eng. C.

[36] Monir T.S.B., Afroz S., Khan R.A., Miah M.Y., Takafuji M., Alam M.A. (2019). pH-sensitive hydrogel from polyethylene oxide and acrylic acid by gamma radiation. J. Compos. Sci..

[37] Elbarbary A.M., Ghobashy M.M. (2017). Controlled release fertilizers using superabsorbent hydrogel prepared by gamma radiation. Radiochim. Acta.

[38] Neves S.C., Moroni L., Barrias C., Granja P. (2019). Leveling Up Hydrogels: Hybrid Systems in Tissue Engineering. Trends Biotechnol..

[39] Zhang M., Zhao X. (2020). Alginate hydrogel dressings for advanced wound management. Int. J. Biol. Macromol..

[40] Lopes J., Fonseca R., Viana T., Fernandes C., Morouço P., Moura C., Biscaia S. (2019). Characterization of Biocompatible Poly(Ethylene Glycol)-Dimethacrylate Hydrogels for Tissue Engineering. Appl. Mech. Mater..

[41] George M., Joseph L., Francis L. (2017). Development and evaluation of silver sulphadiazine loaded sodium alginate gelatin film for wound dressing applications. Eur. J. Pharm. Med. Res..

[42] Abou-Okeil A., Fahmy H., El-Bisi M., Ahmed-Farid O. (2018). Hyaluronic acid/Na-alginate films as topical bioactive wound dressings. Eur. Polym. J..

[43] Rassu G., Salis A., Porcu E.P., Giunchedi P., Roldo M., Gavini E. (2016). Composite chitosan/alginate hydrogel for controlled release of deferoxamine: A system to potentially treat iron dysregulation diseases. Carbohydr. Polym..

[44] García-Cano I., Rocha-Mendoza D., Kosmerl E., Zhang L., Jiménez-Flores R. (2020). Technically relevant enzymes and proteins produced by LAB suitable for industrial and biological activity. Appl. Microbiol. Biotechnol..

[45] Vazquez-Morado L.E., Robles-Zepeda R.E., Ochoa-Leyva A., Arvizu-Flores A.A., Garibay-Escobar A., Castillo-Yañez F., Lopez-Zavala A.A. (2021). Biochemical characterization and inhibition of thermolabile hemolysin from *Vibrio parahaemolyticus* by phenolic compounds. PeerJ.

[46] Horton J., Klarmann-Schulz U., Stephens M., Budge P.J., Coulibaly Y., Debrah A., Debrah L.B., Krishnasastry S., Mwingira U., Ngenya A. (2020). The design and development of a multicentric protocol to investigate the impact of adjunctive doxycycline on the management of peripheral lymphoedema caused by lymphatic filariasis and podo-coniosis. Parasites Vectors.

[47] Bialik-Was K., Malina D., Pluta K. (2020). Sposób Otrzymywania Hydrożelowych Materiałów Opatrunkowych 1AD.

[48] Bahadoran M., Shamloo A., Nokoorani Y.D. (2020). Development of a polyvinyl alcohol/sodium alginate hydrogel-based scaffold incorporating bFGF-encapsulated microspheres for accelerated wound healing. Sci. Rep..

[49] Wang J., Liu C., Shuai Y., Cui X., Nie L. (2014). Controlled release of anticancer drug using graphene oxide as a drug-binding effector in konjac glucomannan/sodium alginate hydrogels. Colloids Surf. B Biointerfaces.

[50] Pereira R., Mendes A., Bártolo P. (2013). Alginate/Aloe Vera Hydrogel Films for Biomedical Applications. Procedia CIRP.

[51] Zhang J., Wang Q., Wang A. (2010). In situ generation of sodium alginate/hydroxyapatite nanocomposite beads as drug-controlled release matrices. Acta Biomater..

[52] De Moura M.R., Guilherme M.R., Campese G.M., Radovanovic E., Rubira A.F., Muniz E.C. (2005). Porous alginate-Ca^2+^ hydrogels interpenetrated with PNIPAAm networks: Interrelationship between compressive stress and pore morphology. Eur. Polym. J..

[53] Pereira R., Tojeira A., Vaz D.B.D.M.C., Mendes A., Bártolo P. (2011). Preparation and Characterization of Films Based on Alginate and Aloe Vera. Int. J. Polym. Anal. Charact..

[54] Koga A.Y., Pereira A.V., Lipinski L., Oliveira M.R. (2017). Evaluation of wound healing effect of alginate films containing Aloe vera (*Aloe barbadensis* Miller) gel. J. Biomater. Appl..

[55] Silva C.L., Pereira J.C., Ramalho A., Pais A.A.C.C., Sousa J.J.S. (2008). Films based on chitosan polyelectrolyte complexes for skin drug delivery: Development and characterization. J. Membr. Sci..

[56] Ghadimi A., Amirilargani M., Mohammadi T., Kasiri N., Sadatnia B. (2014). Preparation of alloyed poly(ether block am-ide)/poly(ethylene glycol diacrylate) membranes for separation of CO_2_/H_2_ (syngas application). J. Memb. Sci..

[57] Huang L., Nishinari K. (2001). Interaction between poly(ethylene glycol) and water as studied by differential scanning calorimetry. J. Polym. Sci. Part B Polym. Phys..

[58] Hatakeyma T., Kasuga H., Tanaka M., Hatakeyama H. (2007). Cold crystallization of poly(ethylene glycol)–water systems. Thermochim. Acta.

[59] Kamoun E.A., Kenawy E.-R., Tamer T.M., El-Meligy M.A., Eldin M.M. (2015). Poly (vinyl alcohol)-alginate physically crosslinked hydrogel membranes for wound dressing applications: Characterization and bio-evaluation. Arab. J. Chem..

[60] Chin S.S., Lyn F.H., Hanani Z.N. (2017). Effect of Aloe vera (*Aloe barbadensis* Miller) gel on the physical and functional properties of fish gelatin films as active packaging. Food Packag. Shelf Life.

